# Survival Benefits of Radiotherapy and Surgery in Lung Cancer Brain Metastases with Poor Prognosis Factors

**DOI:** 10.3390/curroncol30020172

**Published:** 2023-02-13

**Authors:** Lun Liang, Zhenning Wang, Hao Duan, Zhenqiang He, Jie Lu, Xiaobing Jiang, Hongrong Hu, Chang Li, Chengwei Yu, Sheng Zhong, Run Cui, Xiaoyu Guo, Meiling Deng, Yuanyuan Chen, Xiaojing Du, Shaoxiong Wu, Likun Chen, Yonggao Mou

**Affiliations:** 1 Department of Neurosurgery, Sun Yat-sen University Cancer Center, State Key Laboratory of Oncology in South China, Collaborative Innovation Center for Cancer Medicine, Guangzhou 510060, China; 2 Department of Neurosurgery, Dongguan People’s Hospital (Affiliated Dongguan Hospital, South Medical University), Dongguan 523058, China; 3 Department of Neurosurgery, The First Affiliated Hospital of Ji’nan University, Guangzhou 518053, China; 4 Department of Radiotherapy, Sun Yat-sen University Cancer Center, State Key Laboratory of Oncology in South China, Collaborative Innovation Center for Cancer Medicine, Guangzhou 510060, China; 5 Department of Medical Oncology, Sun Yat-sen University Cancer Center, State Key Laboratory of Oncology in South China, Collaborative Innovation Center for Cancer Medicine, Guangzhou 510060, China

**Keywords:** surgery, radiotherapy, poor prognosis, lung cancer brain metastases

## Abstract

Background: Radiotherapy and surgery are the standard local treatments for lung cancer brain metastases (BMs). However, limited studies focused on the effects of radiotherapy and surgery in lung cancer BMs with poor prognosis factors. Methods: We retrospectively analyzed 714 patients with lung cancer BMs. Analyses of overall survival (OS) and risk factors for OS were assessed by the log-rank test and Cox proportional hazard model. Results: Age ≥ 65 years, a Karnofsky Performance Scale (KPS) score ≤ 70, anaplastic large-cell lymphoma kinase (ALK)/epidermal growth factor receptor (EGFR) wild type, and extracranial metastases were related to poor prognosis. Patients were stratified according to these poor prognosis factors. In patients with the ALK/EGFR wild type, whole brain radiotherapy (WBRT), stereotactic radiosurgery (SRS), and surgery improved the OS of patients. WBRT and SRS were the independent protective factors for OS. In patients with extracranial metastases, patients who received WBRT plus SRS or WBRT alone had longer OS than those who did not receive radiotherapy. WBRT plus SRS and WBRT were the independent protective factors for OS. Conclusions: Radiotherapy and surgery are associated with improved survival for lung cancer BMs with the ALK/EGFR wild type. Radiotherapy is associated with improved survival in lung cancer BMs with extracranial metastases.

## 1. Introduction

Lung cancer is the most common primary cancer among patients with brain metastases (BMs), accounting for up to 56% of BMs, and it has high morbidity and limited survival [1]. Surgical resection, stereotactic radiosurgery (SRS), whole brain radiotherapy (WBRT), and systemic therapy are the main treatment modalities for lung cancer BMs and improve the survival and local control of BMs [2,3,4,5]. Recent progress in systemic and local therapies such as targeted therapy, immune checkpoint inhibitors, and radiotherapy, have improved the long-term survival of patients with BMs [6,7]. However, due to the uneven distribution of medical resources in China, advanced systemic treatments such as targeted therapy or immunotherapy are unavailable in many less developed areas. Therefore, traditional standard local therapies, including surgery and radiotherapy, are still important options to treat lung cancer BMs.

A multi-institutional retrospective analysis demonstrated that a Karnofsky Performance Status (KPS) score of 90–100, no extracranial metastases, and epidermal growth factor (EGFR) or anaplastic large-cell lymphoma kinase (ALK) positivity predicted better prognosis in 2186 patients with lung cancer and newly diagnosed BMs [8]. Data from another multi-institutional analysis of 4259 patients with BMs [9] and a phase III trial [10] suggested age < 50 years and limited BMs improved prognosis. However, for patients with poor prognosis, such as low KPS score, older age, EGFR/ALK wild type, and extracranial metastases, few studies have focused on the effects of radiotherapy and surgery. In a III phase, noninferiority randomized trial of 538 patients with non-small-cell lung cancer (NSCLC) with BMs, from 69 UK and three Australian centers, optimal supportive care (OSC) plus WBRT showed no survival benefit over OSC alone in patients with poor prognosis. OSC plus WBRT also showed no survival benefit over OSC alone in patients with an age ≥ 60 years, KPS < 70, extracranial metastases, and multiple BMs [11]. Several studies supported SRS as an optional treatment for older patients with BMs [12,13,14,15]. A previous randomized trial involving 66 patients with single brain metastasis showed that surgery plus WBRT led to better survival than WBRT alone, especially in patients with inactive extracranial metastases [16]. The NCCN guidelines of brain metastases recommended that the treatment of patients with poor prognosis should be individualized, which may include strategies of supportive care, WBRT, SRS, or systemic agents [17].

However, the survival benefit of surgery and radiotherapy in lung cancer brain metastases with poor prognosis is not fully explored. To explore whether standard local treatments, including radiotherapy and surgery, provide survival benefits for patients with lung cancer BMs with poor prognosis factors, we conducted a retrospective and single-center study.

## 2. Materials and Methods

### 2.1. Study Design and Patients

This is a single-center, retrospective, cohort study involving 714 patients diagnosed with lung cancer BMs from 1 January 2004 to 31 December 2019. The study protocol was approved by the local Institutional Review Board and then registered at ClinicalTrials.gov (identifier: NCT05609162). The inclusion criteria were as follows: (1) pathological diagnosis of primary lung cancer; (2) brain metastases confirmed by contrast-enhanced magnetic resonance imaging; (3) availability of complete clinical information; and (4) absence of other malignancies. Finally, 714 patients were eligible for analysis. Clinical information, including age, gender, smoking history, pretreatment KPS, histology, EGFR/ALK status, extracranial metastases, synchronous metastases, location of BMs, number of BMs, surgery, and radiotherapy, was collected from the hospital information system and medical records. EGFR mutation was identified by Real-time PCR, and ALK rearrangement was identified by fluorescence in situ hybridization (FISH). The contrast-enhanced magnetic resonance imaging was used to evaluate the response of the lesions to the various therapies.

This study was approved by the Medical Ethics Committees of SYSUCC (Reference No. B2020-218-01).

### 2.2. Outcome Measurement and Follow-Up

The main outcome was overall survival (OS) after diagnosis of BMs. OS was defined as the time from BM diagnosis to death or censoring at the last follow-up date. The follow-up data were collected by clinical visit and telephone consultation. The follow-up time was from 10 August 2010 to 1 July 2021.

### 2.3. Statistical Analysis

The chi-square test or Fisher’s exact test were used to compare the clinical characteristics between the radiotherapy subgroups (no radiotherapy vs. WBRT alone vs. SRS alone vs. WBRT plus SRS) and surgery subgroups (no surgery vs. surgery) for categorical variables. Kaplan–Meier analysis and the log-rank test were used to compare the difference in OS. Univariate and multivariate Cox proportional hazard regression analyses were used to evaluate risk factors for overall survival. Characteristics with *p* < 0.05 in univariate Cox regression were further analyzed by multivariate Cox regression. Analyses were performed using SPSS version 26.0 (IBM, Armonk, NY, USA) and GraphPad Prism version 8.0 (San Diego, CA, USA, www.graphpad.com (accessed on 17 June 2020)). All statistical tests were two-sided, and *p* < 0.05 was considered statistically significant.

## 3. Results

### 3.1. Clinical Characteristics and Survival Benefits of Surgery and Radiotherapy in All Patients

A total of 714 patients with lung cancer BMs were stratified by radiotherapy and surgery, and their clinical characteristics are shown in Table 1. The number of censored patients was 138 and the number of events was 576. A total of 334 patients received radiotherapy (215 WBRT alone, 79 SRS alone, and 40 WBRT plus SRS), and 380 patients did not receive any radiotherapy. Significantly more patients had lung adenocarcinoma and were treated non-surgically. A total of 211 patients received surgery, and 503 patients did not receive surgery. Significantly more patients were in pretreatment KPS > 70, had lung adenocarcinoma, had the ALK/EGFR mutation, were not synchronous, had extracranial metastases, were supratentorial or multiple, and had no radiotherapy.

**Figure 1:**
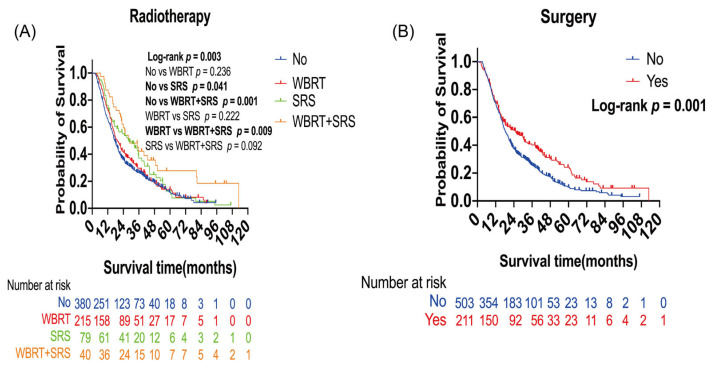
Kaplan–Meier overall survival (OS) curves of patients with lung cancer brain metastases: (**A**) Kaplan–Meier OS curves according to radiotherapy; (**B**) Kaplan–Meier OS curves according to surgery. Plot symbols at both events and censored cases. SRS, stereotactic radiosurgery; WBRT, whole brain radiotherapy.

In survival analysis, patients who received SRS alone (*p* = 0.041) or WBRT plus SRS (*p* = 0.001) showed longer OS than those who did not receive radiotherapy. Median OS was 17.27 months for no radiotherapy, 28.2 months for SRS alone, and 28.87 months for WBRT plus SRS (Figure 1A). Compared with WBRT alone, WBRT plus SRS improved overall survival. Median OS was 19.33 months for WBRT alone and 28.87 months for WBRT plus SRS (*p* = 0.009, Figure 1A). Patients who received surgery showed longer OS than those who did not receive surgery (*p* = 0.001). Median OS was 18.43 months in the non-surgery group and 25.1 months in the surgery group (Figure 1B). In multivariate analyses, WBRT plus SRS (hazard ratio (HR): 0.48; 95% confidence interval (CI): 0.32–0.71) (*p* < 0.001), SRS (HR: 0.70; 95% CI: 0.53–0.92; *p* = 0.01), and surgery (HR: 0.65; 95% CI: 0.53–0.81; *p* < 0.001) were significantly independent protective factors for OS (Table 2).

### 3.2. Survival Benefits of Surgery and Radiotherapy in Patients with Poor Prognosis Factors

Kaplan–Meier analysis and multivariate analysis showed that patients aged ≥ 65 years had shorter OS than those aged < 65 years (*p* = 0.002, Table 2), and an age ≥ 65 years was an independent risk factor for OS (HR: 1.40; 95% CI: 1.13–1.73; *p* = 0.002, Table 2). Patients with a pretreatment KPS score ≤ 70 had shorter OS than those with a pretreatment KPS score > 70 (*p* < 0.001), and a pretreatment KPS score >70 was an independent protective factor for OS (HR: 0.48; 95% CI: 0.36–0.64; *p* < 0.001, Table 2). The ALK/EGFR wild-type group had a shorter OS than the ALK/EGFR mutation group (*p* = 0.008), and the ALK/EGFR mutation was an independent protective factor for OS (HR: 0.74; 95% CI: 0.61–0.89; *p* = 0.002, Table 2). Patients with extracranial metastases had shorter OS than those without extracranial metastases (*p* < 0.001), and extracranial metastases was an independent risk factor for OS (HR: 1.45; 95% CI: 1.21–1.74; *p* < 0.001, Table 2). The results indicated that age ≥ 65 years, pretreatment KPS score ≤ 70, the ALK/EGFR wild type, and extracranial metastases were related to poor prognosis in patients with lung cancer BMs.

The survival benefit of radiotherapy and surgery in patients with poor prognosis factors was further analyzed based on the results above. Patients were stratified according to these poor prognosis factors as subgroups. However, radiotherapy and surgery showed no survival benefit in patients aged ≥ 65 years and with a pretreatment KPS score ≤ 70 (Supplementary Figures S1 and S2).

#### 3.2.1. Survival Benefits of Surgery and Radiotherapy in Patients with Lung Cancer BMs with ALK/EGFR Wild Type

**Figure 2:**
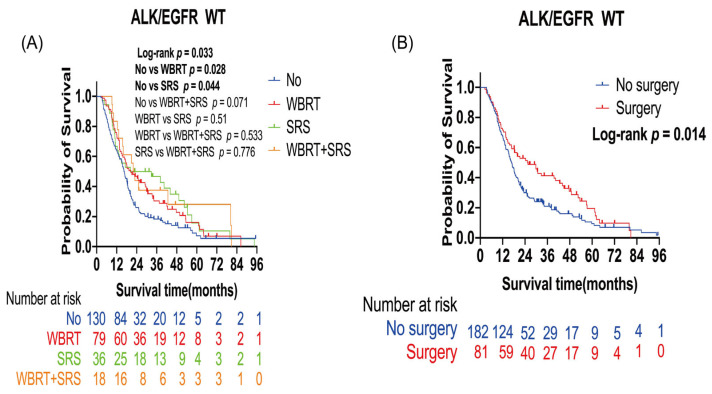
Kaplan–Meier overall survival curves of patients with lung cancer brain metastases with the ALK/EGFR wild type: (**A**) Kaplan–Meier OS curves according to radiotherapy; (**B**) Kaplan–Meier OS curves according to surgery. Plot symbols at both events and censored cases. SRS, stereotactic radiosurgery; WBRT, whole brain radiotherapy; WT, wild type; ALK, anaplastic large-cell lymphoma kinase; EGFR, epidermal growth factor receptor.

The ALK/EGFR wild type led to poor prognosis in patients with lung cancer BMs (Table 2). Further analysis was performed to evaluate the survival benefit of radiotherapy and surgery in these patients. The number of censored patients was 42, and the number of events was 221. A total of 133 patients received radiotherapy (79 WBRT alone, 36 SRS alone, and 18 WBRT plus SRS), and 130 patients did not receive any radiotherapy. A total of 81 patients received surgery, and 182 patients did not receive surgery. Patients with WBRT alone (*p =* 0.028) or SRS alone (*p =* 0.044) had a longer OS than those who did not receive radiotherapy. Median OS was 13.38 months in the non-radiotherapy group, 20.53 months in the WBRT alone group, and 20.33 months in the SRS alone group (Figure 2A). In the multivariate analysis, WBRT alone (HR: 0.67; 95% CI: 0.49–0.92; *p* = 0.01) and SRS alone (HR: 0.65; 95% CI: 0.43–0.99; *p* = 0.04) were the independent protective factors for OS (Table 3). Surgery improved the OS of patients (*p* = 0.014). Median OS was 16.93 months in the non-surgery group and 25.57 months in the surgery group (Figure 2B).

#### 3.2.2. Survival Benefits of Surgery and Radiotherapy in Patients with Lung Cancer BMs with Extracranial Metastases

**Figure 3:**
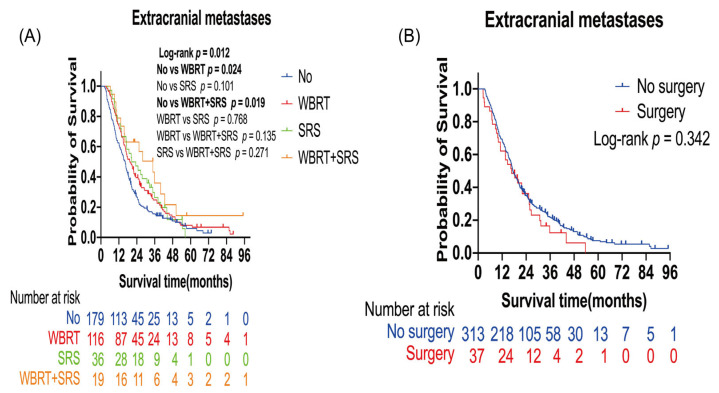
Kaplan–Meier overall survival curves of patients with lung cancer brain metastases with extracranial metastases: (**A**) Kaplan–Meier OS curves according to radiotherapy; (**B**) Kaplan–Meier OS curves according to surgery. Plot symbols at both events and censored cases. SRS, stereotactic radiosurgery; WBRT, whole brain radiotherapy.

Extracranial metastases led to poor prognosis in patients with lung cancer BMs (Table 2). Further analysis was performed to evaluate the effect of radiotherapy and surgery on these patients. The number of censored patients was 41, and the number of events was 309. A total of 171 patients received radiotherapy (116 WBRT alone, 36 SRS alone, and 19 WBRT plus SRS), and 179 patients did not receive any radiotherapy. A total of 37 patients received surgery, and 313 patients did not receive surgery. Patients who received WBRT alone (*p* = 0.024) or WBRT plus SRS (*p* = 0.019) had a longer OS than those who did not receive radiotherapy. Median OS was 16.33 months in the non-radiotherapy group, 19.33 months in the WBRT alone group, and 34.77 months in the WBRT plus SRS group (Figure 3A). In the multivariate analysis, WBRT alone (HR: 0.74; 95% CI: 0.58–0.95; *p* = 0.02) and WBRT plus SRS (HR: 0.50; 95% CI: 0.29–0.87; *p* = 0.01) were the independent protective factors for OS (Table 4).

## 4. Discussion

Our results showed that radiotherapy and surgery both prolonged the OS and predicted good prognosis in patients with lung cancer BMs. An age ≥ 65 years, a pretreatment KPS score ≤ 70, the ALK/EGFR wild type, and extracranial metastases were related to poor prognosis. In patients with the ALK/EGFR wild type, WBRT, SRS, and surgery improved the OS of patients. SRS and WBRT were the independent protective factors for OS. In patients with extracranial metastases, patients who received WBRT plus SRS or WBRT alone had a longer OS than those who did not receive radiotherapy. WBRT plus SRS and WBRT were the independent protective factors for OS.

Previous studies have shown that WBRT plus SRS compared to SRS alone did not improve the survival of patients with BMs [3,18,19,20,21], which was consistent with our results (Figure 1A). Moreover, our results showed no survival benefit of radiation therapy in patients with a pretreatment KPS score ≤ 70, which was also consistent with the latest ASCO (American Society of Clinical Oncology) guidelines for the recommendation that radiotherapy should not be offered to patients with a KPS score < 70 [22]. Patients with extracranial metastases have extensive metastasis and poor prognosis. Our results suggested that WBRT plus SRS was an independent protective factor for OS in patients with lung cancer BMs with extracranial metastases. Besides, WBRT plus SRS provided better survival than non-radiotherapy. The effects of WBRT plus SRS may be related to the improvement of local and distant brain control [21]. The European Association for Neuro-Oncology guidelines recommends that WBRT should be reserved for patients with a high disease burden, such as cancer progression and short life expectancy [23]. More importantly, patients with the ALK/EGFR wild type were often considered insensitive to targeted therapy, and we found that surgery, WBRT, and SRS might help to improve the OS. The findings indicate that radiotherapy and surgery were promising as alternatives to targeted therapy for patients with the ALK/EGFR wild type.

Surgical resection is standard treatment for lung cancer BMs, especially for single BMs [5]. The combination of surgical resection and postoperative radiotherapy was first investigated in patients with BMs [5,24]. Our results indicate that the effect of surgery in patients with lung cancer BMs with poor prognosis factors was limited. Additionally, the baseline variables between the surgery group and the non-surgery group were not balanced, which indicated that patients with single or supratentorial lesions or without extracranial metastases were more likely to undergo surgery in real-world data. In addition, multivariate cox proportional hazard regression analyses were performed to reduce confounding bias. Due to the small number of patients undergoing surgery combined with radiotherapy, we showed the results in Supplementary Figure S3. The significant heterogeneity of patients with lung cancer BMs means that the strategy of local treatment should take the following factors into consideration: the number of BMs, size or location of BMs, symptoms of brain and extracranial disease, and physician preferences.

To the best of our knowledge, this is the first systematic study to investigate the survival benefit of standard local treatments including radiotherapy and surgery in patients with lung cancer BMs with poor prognosis factors. Retrospective analyses have limited reference value due to the impact of observed confounders. All patients were from one single cancer center. Therefore, further studies should focus on multi-institutional prospective trials to validate our results.

## 5. Conclusions

The survival benefit of radiotherapy and surgery in patients with lung cancer BMs with poor prognosis factors varied according to clinical characteristics. The survival benefits of surgery and radiotherapy are limited in patients aged ≥65 years or with a pretreatment KPS score ≤ 70. Radiotherapy and surgery may be alternatives to targeted therapy for patients with the ALK/EGFR wild type. Radiotherapy may provide survival benefits for lung cancer BMs with extracranial metastases.

## Figures and Tables

**Table 1:** Characteristics of patients with lung cancer BMs stratified by surgery or radiotherapy.

Characteristics	Radiotherapy No. (%)					Surgery No. (%)		
	No (*n* = 380)	WBRT (*n* = 215)	SRS (*n* = 79)	WBRT + SRS (*n* = 40)	*p*-Value	No (*n* = 503)	Surgery (*n* = 211)	*p*-Value
Age								
<65 years	310 (81.6)	181 (84.2)	65 (82.3)	32 (80)	0.85	421 (83.7)	167 (79.1)	0.15
≥65 years	70 (18.4)	34 (15.8)	14 (17.7)	8 (20)		82 (16.3)	44 (20.9)	
Gender								
Male	246 (64.7)	122 (56.7)	55 (69.6)	23 (57.5)	0.11	305 (60.6)	141 (66.8)	0.12
Female	134 (35.3)	93 (43.3)	24 (30.4)	17 (42.5)		198 (39.4)	70 (33.2)	
History of smoking								
No	221 (58.2)	129 (60)	42 (53.2)	24 (60)	0.76	290 (57.7)	126 (59.7)	0.61
Yes	159 (41.8)	86 (40)	37 (46.8)	16 (40)		213 (42.3)	85 (40.3)	
Pretreatment KPS								
≤70	39 (10.3)	18 (8.4)	5 (6.3)	0 (0)	0.11	24 (4.8)	38 (18)	<0.001
>70	341 (89.7)	197 (91.6)	74 (93.7)	40 (100)		479 (95.2)	173 (82)	
Histology								
LUAD	324 (85.3)	198 (92.1)	64 (81)	32 (80)	0.02	454 (90.3)	164 (77.7)	<0.001
Others	56 (14.7)	17 (7.9)	15 (19)	8 (20)		49 (9.7)	47 (22.3)	
ALK/EGFR status								
WT	130 (34.2)	79 (36.7)	36 (45.6)	18 (45)	0.40	182 (36.2)	81 (38.4)	<0.001
MT	165 (43.4)	94 (43.7)	30 (38)	17 (42.5)		241 (47.9)	65 (30.8)	
NOS	85 (22.4)	42 (19.5)	13 (16.5)	5 (12.5)		80 (15.9)	65 (30.8)	
Synchronous metastases								
No	242 (63.7)	145 (67.4)	52 (65.8)	31 (77.5)	0.33	380 (75.5)	90 (42.7)	<0.001
Yes	138 (36.3)	70 (32.6)	27 (34.2)	9 (22.5)		123 (24.5)	121 (57.3)	
Extracranial metastases								
No	201 (52.9)	99 (46)	43 (54.4)	21 (52.5)	0.38	190 (37.8)	174 (82.5)	<0.001
Yes	179 (47.1)	116 (54)	36 (45.6)	19 (47.5)		313 (62.2)	37 (17.5)	
Location of BMs								
Supratentorial	292 (76.8)	162 (75.3)	60 (75.9)	28 (70)	0.83	381 (75.7)	161 (76.3)	<0.001
Subtentorial	19 (5)	12 (5.6)	5 (6.3)	1 (2.5)		14 (2.8)	23 (10.9)	
Both	69 (18.2)	41 (19.1)	14 (17.7)	11 (27.5)		108 (21.5)	27 (12.8)	
Number of BMs								
Single	161 (42.4)	82 (38.1)	30 (38)	16 (40)	0.78	146 (29)	143 (67.8)	<0.001
Multiple	219 (57.6)	133 (61.9)	49 (62)	24 (60)		357 (71)	68 (32.2)	
Surgery								
No	248 (65.3)	161 (74.9)	59 (74.7)	35 (87.5)	0.004			
Yes	132 (34.7)	54 (25.1)	20 (25.3)	5 (12.5)				
Radiotherapy	/							
No						248 (49.3)	132 (62.6)	0.004
WBRT						161 (32)	54 (25.6)	
SRS						59 (11.7)	20 (9.5)	
WBRT + SRS						35 (7)	5 (2.4)	

Abbreviations: BMs, brain metastases; multiple, number of BMs ≥ 2; LUAD, lung adenocarcinoma; MT, mutation; NOS, unknown or untested; SRS, stereotactic radiosurgery; WBRT, whole brain radiotherapy; WT, wild type; ALK, anaplastic large-cell lymphoma kinase; EGFR, epidermal growth factor receptor.

**Table 2:** Prognostic analysis for patients with lung cancer BMs.

Characteristics	Level	Median OS (Month)	Logrank *p*-Value	Univariate Cox Regression		Multivariate Cox Regression	
				HR (95% CI)	*p*-Value	HR (95% CI)	*p*-Value
Age	<65 years	20.37	0.002	Ref		Ref	
	≥65 years	15.67		1.39 (1.13–1.72)	0.002	1.40 (1.13–1.73)	0.002
Gender	Male	18.2	0.44	Ref			
	Female	20.57		0.94 (0.79–1.11)	0.44		
History of smoking	No	20.6	0.06	Ref			
	Yes	17.07		1.17 (0.99–1.38)	0.061		
Pretreatment KPS	≤70	11.83	<0.001	Ref		Ref	
	>70	20.37		0.49 (0.37–0.65)	<0.001	0.48 (0.36–0.64)	<0.001
Histology	LUAD	20.23	0.04	Ref		Ref	
	Others	16.7		1.29 (1.02–1.64)	0.04	1.38 (1.07–1.78)	0.01
EGFR/ALK status	WT	18.1		Ref		Ref	
	MT	23.67	0.008	0.78 (0.65–0.94)	0.009	0.74 (0.61–0.89)	0.002
Synchronous metastases	No	19.57	0.75	Ref			
	Yes	18.1		1.03 (0.87–1.22)	0.75		
Extracranial metastases	No	22.07	<0.001	Ref		Ref	
	Yes	17.4		1.47 (1.25–1.74)	<0.001	1.45 (1.21–1.74)	<0.001
Location of BMs	Supratentorial	18.87	Supratentorial vs. Subtentorial 0.45	Ref			
	Subtentorial	18.7	Supratentorial vs. Both 0.93	0.87 (0.60–1.26)	0.46		
	Both	20.87	Subtentorial vs. Both 0.53	0.99 (0.80–1.22)	0.92		
Number of BMs	Single	20.17	0.08	Ref			
	Multiple	18.5		1.16 (0.98–1.37)	0.08		
Radiotherapy	No	17.27	No vs. WBRT 0.24	Ref		Ref	
	WBRT	19.33	No vs. SRS 0.04	0.89 (0.74–1.07)	0.22	0.84 (0.69–1.01)	0.06
	SRS	28.2	No vs. WBRT + SRS 0.001	0.75 (0.57–0.99)	0.4	0.70 (0.53–0.92)	0.01
	WBRT + SRS	28.87	WBRT vs. SRS 0.222	0.51 (0.35–0.76)	0.01	0.48 (0.32–0.71)	<0.001
			WBRT vs. WBRT + SRS 0.009				
			SRS vs. WBRT + SRS 0.092				
Surgery	No	18.43	0.001	Ref		Ref	
	Yes	25.1		0.73 (0.61–0.88)	0.001	0.65 (0.53–0.81)	<0.001

**Table 3:** Univariate and multivariate analyses of risk factors for OS in lung cancer BMs patients with the ALK/EGFR wild type.

Covariates	Level	Univariate Analysis		Multivariate Analysis	
		HR (95% CI)	*p*-Value	HR (95% CI)	*p*-Value
Age	<65 years	Ref			
	≥65 years	1.17 (0.84–1.63)	0.34		
Gender	Male	Ref			
	Female	0.95 (0.70–1.29)	0.74		
History of smoking	No	Ref			
	Yes	1.06 (0.82–1.39)	0.65		
Pretreatment KPS	≤70	Ref			
	>70	0.79 (0.49–1.29)	0.35		
History of smoking	LUAD	Ref			
	Others	1.24 (0.87–1.76)	0.24		
Synchronous metastases	No	Ref			
	Yes	0.79 (0.69–1.05)	0.10		
Extracranial metastases	No	Ref		Ref	
	Yes	1.56 (1.20–2.05)	0.001	1.44 (1.07–1.94)	0.02
Location of BMs	Supratentorial	Ref			
	Subtentorial	0.74 (0.43–1.28)	0.29		
	Both	1.51 (1.08–2.12)	0.17		
Number of BMs	Single	Ref			
	Multiple	1.22 (0.93–1.59)	0.15		
Radiotherapy	No	Ref		Ref	
	WBRT	0.72 (0.53–0.97)	0.03	0.67 (0.49–0.92)	0.01
	SRS	0.64 (0.43–0.97)	0.03	0.65 (0.43–0.99)	0.04
	WBRT + SRS	0.60 (0.34–1.04)	0.07	0.60 (0.34–1.05)	0.07
Surgery	No	Ref		Ref	
	Yes	0.69 (0.52–0.93)	0.02	0.78 (0.56–1.08)	0.14

**Table 4:** Univariate and multivariate analyses of risk factors for OS in lung BMs patients with extracranial metastases.

Covariates	Level	Univariate Analysis		Multivariate Analysis	
		HR (95% CI)	*p*-Value	HR (95% CI)	*p*-Value
Age	<65 years	Ref		Ref	
	≥65 years	1.44 (1.07–1.95)	0.02	1.45 (1.07–2.00)	0.02
Gender	Male	Ref			
	Female	0.94 (0.75–1.18)	0.59		
History of smoking	No	Ref			
	Yes	1.17 (0.93–1.47)	0.14		
Pretreatment KPS	≤70	Ref		Ref	
	>70	0.48 (0.30–0.75)	0.001	0.53 (0.33–0.85)	0.008
Histology	LUAD	Ref			
	Others	1.27 (0.87–1.86)	0.22		
Synchronous metastases	No	Ref			
	Yes	1.23 (0.96–1.54)	0.11		
ALK/EGFR status	WT	Ref		Ref	
	MT	0.71 (0.55–0.91)	0.006	0.73 (0.57–0.94)	0.02
	NOS	1.29 (0.93–1.80)	0.13	1.32 (0.95–1.84)	0.10
Location of BMs	Supratentorial	Ref			
	Subtentorial	1.34 (0.73–2.46)	0.34		
	Both	0.79 (0.60–1.05)	0.10		
Number of BMs	Single	Ref			
	Multiple	0.93 (0.72–1.19)	0.55		
Radiotherapy	No	Ref		Ref	
	WBRT	0.73 (0.59–0.96)	0.03	0.74 (0.58–0.95)	0.02
	SRS	0.71 (0.49–1.04)	0.08	0.72 (0.49–1.06)	0.09
	WBRT + SRS	0.50 (0.29–0.86)	0.01	0.50 (0.29–0.87)	0.01
Surgery	No	Ref			
	Yes	1.20 (0.83–1.73)	0.34		

## Data Availability

The datasets are available from the corresponding author on reasonable request.

## Supplementary Materials

The following supporting information can be downloaded at: https://www.mdpi.com/article/10.3390/curroncol30020172/s1, Figure S1. Kaplan–Meier overall survival (OS) curves of patients with lung cancer brain metastases with age ≥65 years.; Figure S2. Kaplan–Meier overall survival (OS) curves of patients with lung cancer brain metastases with pretrement KPS ≤70. Figure S3. Kaplan–Meier overall survival (OS) curves of patients with lung cancer brain metastases of all subgroups and all treatment groups.

## References

[1] Achrol A.S., Rennert R.C., Anders C., Soffietti R., Chang S.D. (2019). Brain metastases. Nat. Rev. Dis. Prim..

[2] Goldberg S.B., Contessa J.N., Omay S.B., Chiang V. (2015). Lung Cancer Brain Metastases. Cancer J..

[3] Kocher M., Soffietti R., Abacioglu U., Villà S., Fauchon F., Baumert B.G., Fariselli L., Tzuk-Shina T., Kortmann R.-D., Carrie C. (2011). Adjuvant Whole-Brain Radiotherapy Versus Observation After Radiosurgery or Surgical Resection of One to Three Cerebral Metastases: Results of the EORTC 22952–26001 Study. J. Clin. Oncol..

[4] Mahajan A., Ahmed S., McAleer M.F., Weinberg J.S., Li J., Brown P., Settle S., Prabhu S.S., Lang F.F., Levine N. (2017). Post-operative stereotactic radiosurgery versus observation for completely resected brain metastases: A single-centre, randomised, controlled, phase 3 trial. Lancet Oncol..

[5] Patchell R.A., Tibbs P.A., Walsh J.W., Dempsey R.J., Maruyama Y., Kryscio R.J., Markesbery W.R., Macdonald J.S., Young B. (1990). A Randomized Trial of Surgery in the Treatment of Single Metastases to the Brain. N. Engl. J. Med..

[6] Chamberlain M.C., Baik C.S., Gadi V.K., Bhatia S., Chow L.Q. (2017). Systemic therapy of brain metastases: Non–small cell lung cancer, breast cancer, and melanoma. Neuro-Oncology.

[7] Gogineni E., Vargo J.A., Glaser S.M., Flickinger J.C., Burton S.A., Engh J.A., Amankulor N.M., Beriwal S., Quinn A.E., Ozhasoglu C. (2017). Long-Term Survivorship Following Stereotactic Radiosurgery Alone for Brain Metastases: Risk of Intracranial Failure and Implications for Surveillance and Counseling. Neurosurgery.

[8] Sperduto P.W., Yang T.J., Beal K., Pan H., Brown P.D., Bangdiwala A., Shanley R., Yeh N., Gaspar L.E., Braunstein S. (2017). Estimating Survival in Patients with Lung Cancer and Brain Metastases An Update of the Graded Prognostic Assessment for Lung Cancer Using Molecular Markers (Lung-molGPA). JAMA Oncol..

[9] Sperduto P.W., Chao S.T., Sneed P.K., Luo X., Suh J., Roberge D., Bhatt A., Jensen A.W., Brown P.D., Shih H. (2010). Diagnosis-Specific Prognostic Factors, Indexes, and Treatment Outcomes for Patients with Newly Diagnosed Brain Metastases: A Multi-Institutional Analysis of 4259 Patients. Int. J. Radiat. Oncol..

[10] Sahgal A., Aoyama H., Kocher M., Neupane B., Collette S., Tago M., Shaw P., Beyene J., Chang E.L. (2015). Phase 3 Trials of Stereotactic Radiosurgery with or without Whole-Brain Radiation Therapy for 1 to 4 Brain Metastases: Individual Patient Data Meta-Analysis. Int. J. Radiat. Oncol..

[11] Mulvenna P., Nankivell M., Barton R., Faivre-Finn C., Wilson P., McColl E., Moore B., Brisbane I., Ardron D., Holt T. (2016). Dexamethasone and supportive care with or without whole brain radiotherapy in treating patients with non-small cell lung cancer with brain metastases unsuitable for resection or stereotactic radiotherapy (QUARTZ): Results from a phase 3, non-inferiority, randomised trial. Lancet.

[12] Chen L., Shen C., Redmond K.J., Page B., Kummerlowe M., Mcnutt T., Bettegowda C., Rigamonti D., Lim M., Kleinberg L. (2017). Use of Stereotactic Radiosurgery in Elderly and Very Elderly Patients with Brain Metastases to Limit Toxicity Associated with Whole Brain Radiation Therapy. Int. J. Radiat. Oncol. Biol. Phys..

[13] Higuchi Y., Yamamoto M., Serizawa T., Sato Y., Shuto T., Akabane A., Jokura H., Yomo S., Nagano O., Kawagishi J. (2019). Stereotactic radiosurgery in elderly patients with brain metastases: Comparison with non-elderly patients using database of a multi-institutional prospective observational study (JLGK0901-Elderly). J. Neuro-Oncol..

[14] Minniti G., Esposito V., Clarke E., Scaringi C., Bozzao A., Lanzetta G., De Sanctis V., Valeriani M., Osti M., Enrici R.M. (2013). Stereotactic radiosurgery in elderly patients with brain metastases. J. Neuro-Oncol..

[15] Yomo S., Hayashi M. (2016). Is upfront stereotactic radiosurgery a rational treatment option for very elderly patients with brain metastases? A retrospective analysis of 106 consecutive patients age 80 years and older. BMC Cancer.

[16] Noordijk E.M., Vecht C.J., Haaxma-Reiche H., Padberg G.W., Voormolen J.H., Hoekstra F.H., Tans J.T., Lambooij N., Metsaars J.A., Wattendorff A. (1994). The choice of treatment of single brain metastasis should be based on extracranial tumor activity and age. Int. J. Radiat. Oncol..

[17] Nabors L.B., Portnow J., Ahluwalia M., Baehring J., Darlow S.D. (2020). Central Nervous System Cancers, Version 3.2020, NCCN Clinical Practice Guidelines in Oncology. J. Natl. Compr. Cancer Netw. JNCCN.

[18] Aoyama H., Shirato H., Tago M., Nakagawa K., Toyoda T., Hatano K., Kenjyo M., Oya N., Hirota S., Shioura H. (2006). Stereotactic Radiosurgery Plus Whole-Brain Radiation Therapy vs Stereotactic Radiosurgery Alone for Treatment of Brain Metastases: A randomized controlled trial. JAMA.

[19] Brown P.D., Jaeckle K., Ballman K.V., Farace E., Cerhan J.H., Anderson S.K., Carrero X.W., Barker F.G., Deming R., Burri S.H. (2016). Effect of Radiosurgery Alone vs Radiosurgery with Whole Brain Radiation Therapy on Cognitive Function in Patients with 1 to 3 Brain Metastases: A Randomized Clinical Trial. JAMA.

[20] Chang E.L., Wefel J.S., Hess K.R., Allen P.K., Lang F.F., Kornguth D.G., Arbuckle R.B., Swint J.M., Shiu A.S., Maor M.H. (2009). Neurocognition in patients with brain metastases treated with radiosurgery or radiosurgery plus whole-brain irradiation: A randomised controlled trial. Lancet Oncol..

[21] Tsao M.N., Xu W., Wong R.K., Lloyd N., Laperriere N., Sahgal A., Rakovitch E., Chow E. (2018). Whole brain radiotherapy for the treatment of newly diagnosed multiple brain metastases. Cochrane Database Syst. Rev..

[22] Vogelbaum M.A., Brown P.D., Messersmith H., Brastianos P.K., Burri S., Cahill D., Dunn I.F., Gaspar L.E., Gatson N.T.N., Gondi V. (2022). Treatment for Brain Metastases: ASCO-SNO-ASTRO Guideline. J. Clin. Oncol..

[23] Soffietti R., Abacioglu U., Baumert B., Combs S.E., Kinhult S., Kros J.M., Marosi C., Metellus P., Radbruch A., Freixa S.S.V. (2017). Diagnosis and treatment of brain metastases from solid tumors: Guidelines from the European Association of Neuro-Oncology (EANO). Neuro-Oncology.

[24] Patchell R.A., Tibbs P.A., Regine W.F., Dempsey R.J., Mohiuddin M., Kryscio R.J., Markesbery W.R., Foon K.A., Young B. (1998). Postoperative Radiotherapy in the Treatment of Single Metastases to the Brain: A randomized trial. JAMA.

