# Supplementary material for: Survival Benefits of Radiotherapy and Surgery in Lung Cancer Brain Metastases with Poor Prognosis Factors
Source: Curr Oncol. 2023 Feb 13;30(2):2227–36. doi: 10.3390/curroncol30020172 (PMC9954973; doi:10.3390/curroncol30020172)
Supplement: Supplementary file 1 [file curroncol-30-00172-s001.zip › curroncol-2116433-supplementary.pdf]

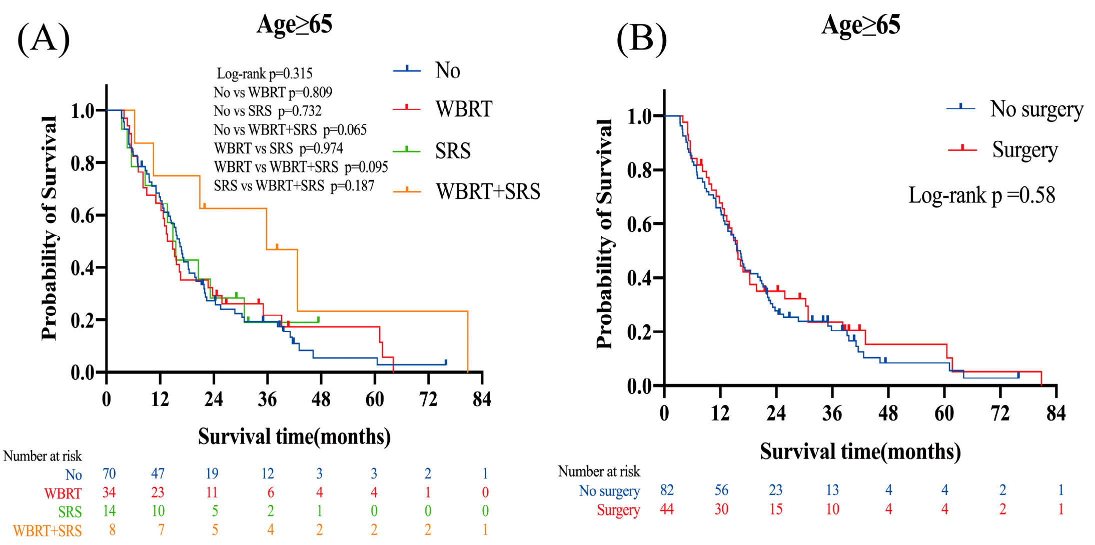

Figure S1. Kaplan–Meier overall survival (OS) curves of patients with lung cancer brain metastases with age ≥65 years. (**A**) Kaplan–Meier OS according to radiotherapy. (**B**) Kaplan–Meier OS according to surgery.

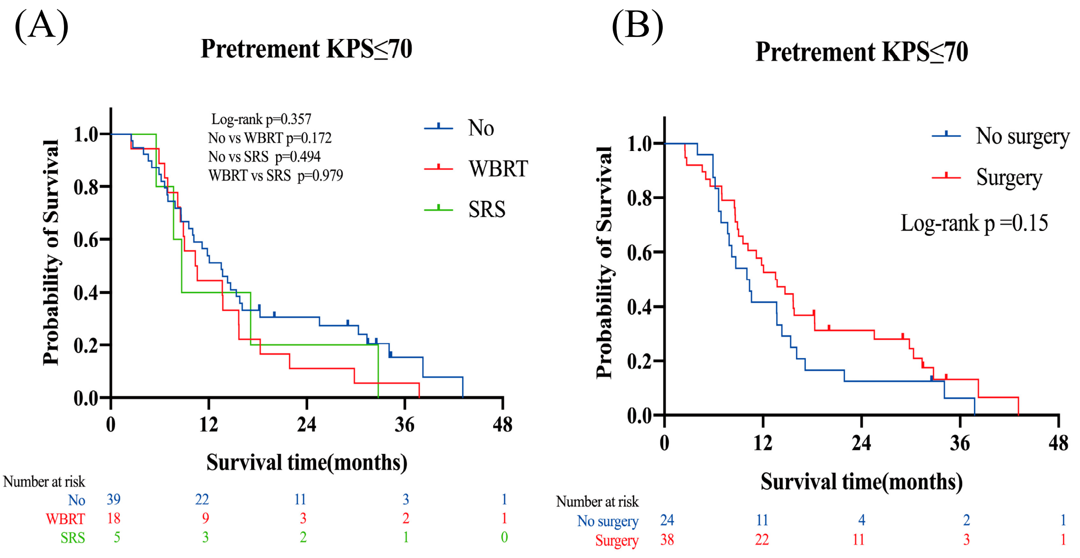

Figure S2. Kaplan–Meier overall survival (OS) curves of patients with lung cancer brain metastases with pretrement KPS ≤70. (**A**) Kaplan–Meier OS according to radiotherapy. (**B**) Kaplan–Meier OS curves according to surgery.

Age≥65

(A)

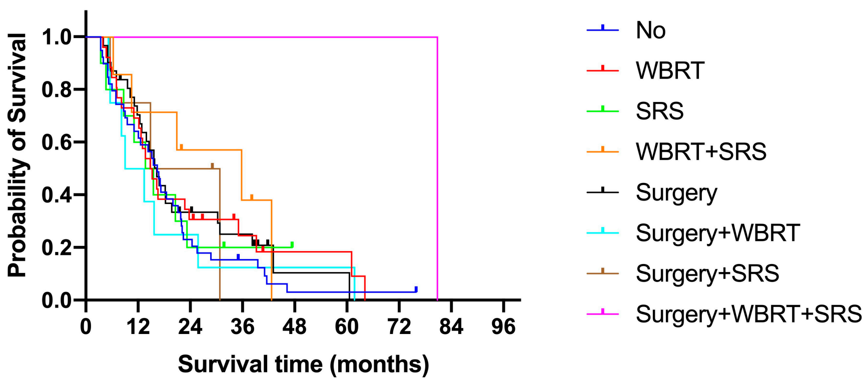

Number at risk								
No	39	26	10	6	2	2	2	1
WBRT	26	19	9	5	3	3	1	0
SRS	10	7	3	2	1	1	0	0
WBRT+SRS	7	6	4	3	1	1	0	0
Surgery	31	22	10	7	2	2	1	0
Surgery+WBRT	8	5	3	2	2	2	1	0
Surgery+SRS	4	4	3	1	0	0	0	0
Surgery+WBRT+SRS	1	1	1	1	1	1	1	1

Pretrement KPS≤70

(B)

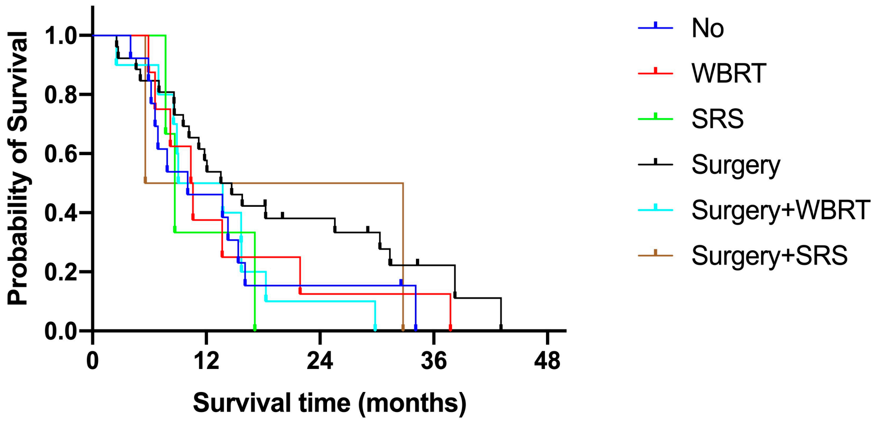

Number at risk					
No	13	7	3	1	0
WBRT	8	4	2	2	1
SRS	3	2	1	0	0
Surgery	26	16	9	3	1
Surgery+WBRT	10	6	2	1	0
Surgery+SRS	2	2	2	1	0

(C)

ALK/EGFR WT

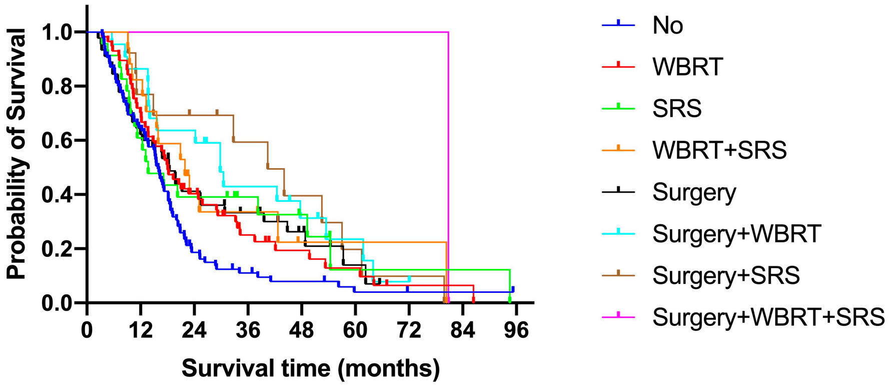

Number at risk

No	85	56	16	9	6	3	2	2	1
WBRT	57	41	22	11	7	5	2	2	1
SRS	23	15	10	7	5	2	2	2	1
WBRT+SRS	17	15	7	5	2	2	2	1	0
Surgery	45	29	17	12	7	3	1	0	0
Surgery+WBRT	22	20	15	9	6	4	2	1	0
Surgery+SRS	13	11	9	7	5	3	2	1	0
Surgery+WBRT+SRS	1	1	1	1	1	1	1	1	0

No vs WBRT p=0.033
No vs SRS p=0.044
No vs Surgery p=0.035
No vs Surgery+WBRT p=0.002
No vs Surgery+SRS p=0.009

Extracranial metastases

(D)

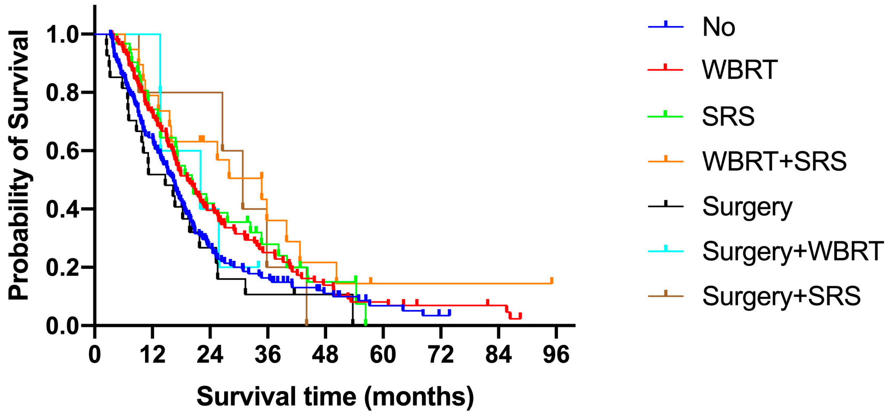

Number at risk

No	152	99	40	23	12	5	2	1	0
WBRT	11	82	43	24	13	8	5	4	1
SRS	31	24	14	8	4	1	0	0	0
WBRT+SRS	19	16	11	7	4	2	2	2	1
Surgery	27	15	6	3	2	1	0	0	0
Surgery+WBRT	5	5	3	1	0	0	0	0	0
Surgery+SRS	5	5	5	3	1	0	0	0	0

No vs WBRT p=0.048
No vs WBRT+SRS p=0.024
WBRT+SRS vs Surgery p=0.021

Figure S3. Kaplan–Meier overall survival (OS) curves of patients with lung cancer brain metastases of all subgroups and all treatment groups.
